# Spatial Distribution Patterns of Root-Associated Bacterial Communities Mediated by Root Exudates in Different Aged Ratooning Tea Monoculture Systems

**DOI:** 10.3390/ijms18081727

**Published:** 2017-08-08

**Authors:** Yasir Arafat, Xiaoya Wei, Yuhang Jiang, Ting Chen, Hafiz Sohaib Ahmed Saqib, Sheng Lin, Wenxiong Lin

**Affiliations:** 1 Key Laboratory of Fujian Province for Agroecological Process and Safety Monitoring, Fujian Agriculture and Forestry University, Fuzhou 35002, China; arafat_pep@yahoo.com (Y.A.); xlwei112930@163.com (X.W.); janmiky@163.com (Y.J.); iamchenting@126.com (T.C.); 2 Key Laboratory of Ministry of Education for Crop Genetics/Breeding and Integrative Utilization, Fujian Agriculture and Forestry University, Fuzhou 35002, China; 3 College of Life Science, Fujian Agriculture and Forestry University, Fuzhou 35002, China; 4 Institute of Agroecological Ecology, Fujian Agriculture and Forestry University, Fuzhou 35002, China; sohaibsaqib@gmail.com

**Keywords:** monoculture, allelochemicals, microbiomes, rhizo-compartments, high-throughput sequence, redundancy analysis (RDA), high performance liquid chromatography-electrospray ionization-mass spectrometry (HPLC–ESI–MS)

## Abstract

Positive plant–soil feedback depends on beneficial interactions between roots and microbes for nutrient acquisition; growth promotion; and disease suppression. Recent pyrosequencing approaches have provided insight into the rhizosphere bacterial communities in various cropping systems. However; there is a scarcity of information about the influence of root exudates on the composition of root-associated bacterial communities in ratooning tea monocropping systems of different ages. In Southeastern China; tea cropping systems provide the unique natural experimental environment to compare the distribution of bacterial communities in different rhizo-compartments. High performance liquid chromatography–electrospray ionization–mass spectrometry (HPLC–ESI–MS) was performed to identify and quantify the allelochemicals in root exudates. A high-throughput sequence was used to determine the structural dynamics of the root-associated bacterial communities. Although soil physiochemical properties showed no significant differences in nutrients; long-term tea cultivation resulted in the accumulation of catechin-containing compounds in the rhizosphere and a lowering of pH. Moreover; distinct distribution patterns of bacterial taxa were observed in all three rhizo-compartments of two-year and 30-year monoculture tea; mediated strongly by soil pH and catechin-containing compounds. These results will help to explore the reasons why soil quality and fertility are disturbed in continuous ratooning tea monocropping systems; and to clarify the associated problems.

## 1. Introduction

Plant–soil feedback is a two-step process in which the presence of a plant alters the structure and composition of the rhizosphere microorganism community, and then that change in the microorganism community alters the growth rate and development of the plant [1,2,3,4,5,6]. In rhizosphere soil there exists a positive soil feedback mechanism, in which microorganisms mediate plant efficiency by producing plant growth hormones, promoting plant nutrient uptake, competitively suppressing plant pathogens, and fixing nitrogen [7]. On the other hand, a negative plant–soil feedback also exists, caused by the accumulation of phytotoxic compounds in soil. This feedback inhibits beneficial microbes but promotes parasites and pathogen outbreaks, and in turn results in autotoxicity or soil sickness, and the hindrance of plant growth and development, which reduces plant yield and quality [8,9,10]. Autotoxicity and soil sickness are the typical results of negative plant–soil interactions, mainly driven by agricultural landscape simplification, such as continuous monoculture which is the cropping of the same plant in the same field for many consecutive years [11,12,13,14]. Production of autotoxins, soil nutrient imbalance, and the alteration of soil-associated microbial community composition are considered as fundamental issues related to autotoxicity and soil sickness [15,16].

Autotoxicity-related problems are observed when practicing consecutive monoculture of agronomic crops, forages, fruits, medicinal, and horticultural plants [6,13,14,16,17,18,19,20,21,22]. Several groups of chemicals have been identified for instigating autotoxicity, such as steroids, flavonoids, cyanogenic glycosides, alkaloids, terpenoids, and phenolic acids. Among these autotoxins, various flavonoids are known for their antimicrobial activity [23,24,25]. Catechin, one of the major flavonoids, is widely reported in wine, traditional Chinese medicine, green tea, natural fruits [26,27,28,29], and also in invasive plant species such as *Centuria muculusa* [30,31] and *Rhododendron formosanum* [32]. Studies have also shown that catechins play a vital role in the successful invasion and establishment of invasive species due to its allelopathic action [32,33], but the influential role of catechins in causing problems, by altering the distribution of microbes associated with modern monocropping systems, especially in consecutive ratooning tea gardens, has not yet been studied.

*Camellia sinensis* (L.) O. Kuntze is one of the most economically valuable mountainous crops in Southern China and is also widely grown in Africa and Asia. It has been commercially grown for almost 1500 years in China. However, the problems related to tea cultivation, such as reduction of tea crop productivity and quality, have increased overtime after the establishment of tea plants in the same soil for several years [34,35]. Researchers found that tea leaves and roots produce various biologically active catechins, which can act as allelochemicals [25,36,37]. Studies also proposed that catechins appear to degrade quickly into other chemical compounds after introduction into the soil, which can affect the population dynamics and growth of several bacterial taxa [38,39]. Most studies addressing the problems related to continuous tea monocropping systems have simply investigated the numerical responses of microbial communities mediated by the soil physiochemical properties [35,36]. Moreover, the influence of root exudates on the distribution of rhizosphere bacterial communities in a continuous monocultured tea garden has not been studied. This study was designed to explore the distribution of bacterial taxa present in the rhizosphere of new and continuous monocropping tea systems and an adjacent uncultivated field. We also wanted to investigate the influence of root exudates and soil physiochemical properties on the abundance of those bacterial taxa. Specifically, we hypothesized that different lengths of time of ratooning tea monoculture fields share bacteria to varying degrees and potentially this would be mediated over time by the variations in soil physiochemical properties and root exudates.

## 2. Results

### 2.1. The Problems of Camellia sinensis (L.) Plantation under Continuous Monoculture

When compared to a new tea field planted two years ago, the tea field which was continuously monocultured for 30 years showed poor growth, chlorosis, wilting, and ratooning problems (Figure S1a,b). Moreover, the quality of tea leaves was significantly lower in the continuously monocultured tea fields than in the newly planted tea garden (Table 1).

### 2.2. Soil Physio-Chemical Properties and Root Exudates

**Figure 1:**
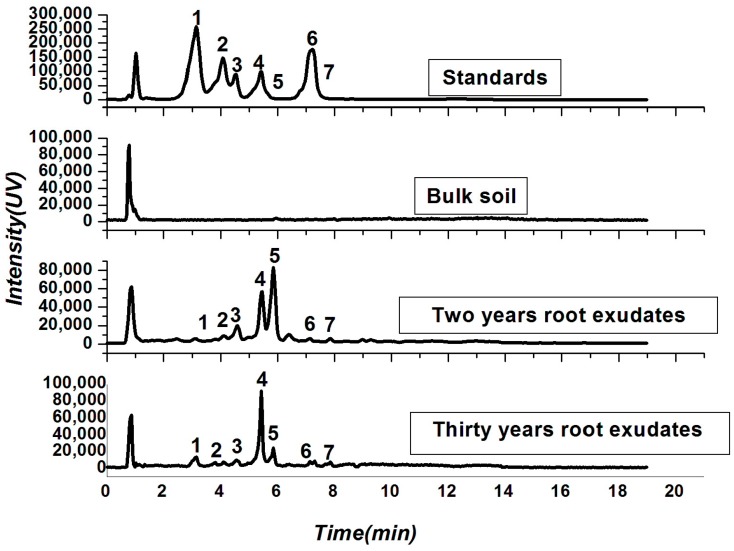
High performance liquid chromatography–electrospray ionization–mass spectrometry (HPLC–ESI–MS) spectra of catechins in root exudates collected from different plantations of different ages tea fields and bulk soil; “1”represents protocatechuic acid (PCA) with a retention time of 3.12 min; “2”represents epigallocatechin (EGC) with a retention time of 4.07 min; “3” represents epigallocatechin-3-gallate (EGCG) with a retention time of 4.53 min; “4” represents epicatechin (EC) with a retention time of 5.43 min; “5” represents (+)-catechin (C) with a retention time of 5.72 min; “6” represents epicatechin-3-gallate (ECG) with a retention time of 7.13 min; and “7” represents taxifolin (TF) with a retention time of 7.32 min.

Soil physio-chemical properties and root exudates were analyzed to determine the main impacts of tea monoculture in a field. Most of the soil nutrients, including nitrogen (N), phosphorus (P), and potassium (K), did not show a significant difference in bulk soil between the newly planted tea field and the 30-year continuously monocultured tea field, but soil pH was significantly lower in the 30-year-old tea plantation compared to the bulk soil in the two-year tea plantation (Table S1). Meanwhile, soil moisture in the 30-year-old tea field was higher in the bulk soil than in the two-year-old tea plantation (Table S1). Seven compounds including protocatechuic acid (PCA), epigallocatechin (EGC), epigallocatechin gallate (EGCG), epicatechin (EC), (+)-catechin (C), epicatechingallate (ECG), and taxifolin (TF) were identified in the root exudates in both the newly planted tea field and the 30-year continuously monocultured tea field (Figure 1). Moreover, in the young plantation, the concentrations of PCA, EGC, EGCG, EC, C, ECG, and TF were 2.04, 1.73, 6.95, 11.98, 21.31, 1.18, and 1.61 mg/kg, respectively. Meanwhile in the 30-year continuous monoculture field, these concentrations were 14.58, 3.20, 3.27, 21.38, 3.70, 2.62, and 3.65 mg/kg, respectively. The recovery percentage of PCA, EGC, EGCG, EC, C, ECG, and TF were in an acceptable range of between 60% and 120% (Table S2).

### 2.3. 16S rDNA-Based Meta-Genomic Analysis of Tea Root-Associated Bacteria

**Figure 2:**
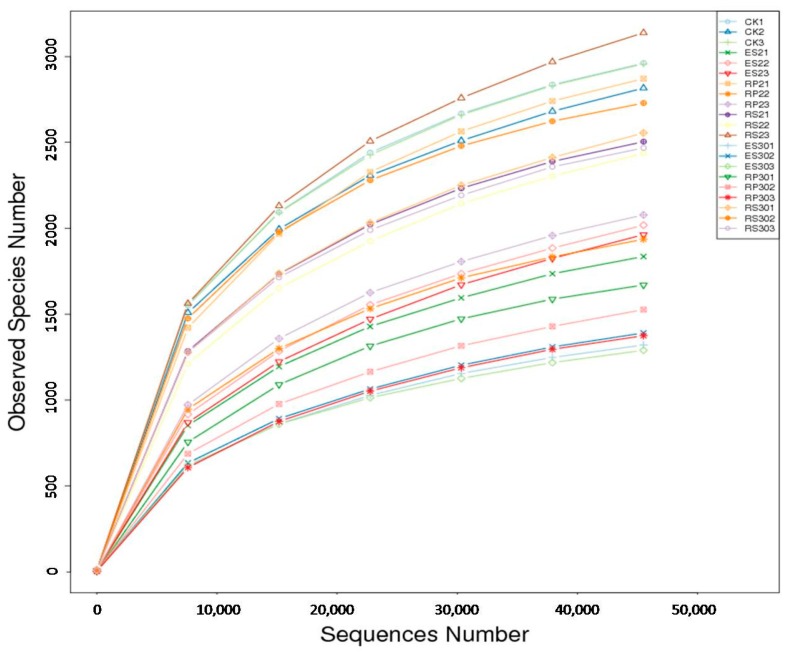
The rarefaction curve based on 97% similarity. CK1, CK2, CK3 refer to the bulk soil, RS21, RS22, RS23 represent rhizosphere of the two-year-old tea plantation, RP21, RP22, RP23 represent rhizoplane of the two-year-old tea plantation, and ES21, ES22, ES23 represent endosphere of the two-year-old tea plantation respectively. While RS301, RS302, RS303 represent rhizosphere of the thirty-year-old tea plantation, RP301, RP302, RP303 represent rhizoplane of the thirty-year-old tea plantation, and ES301, ES302, ES303 represent endosphere of the thirty-year-old tea plantation respectively.

**Figure 3:**
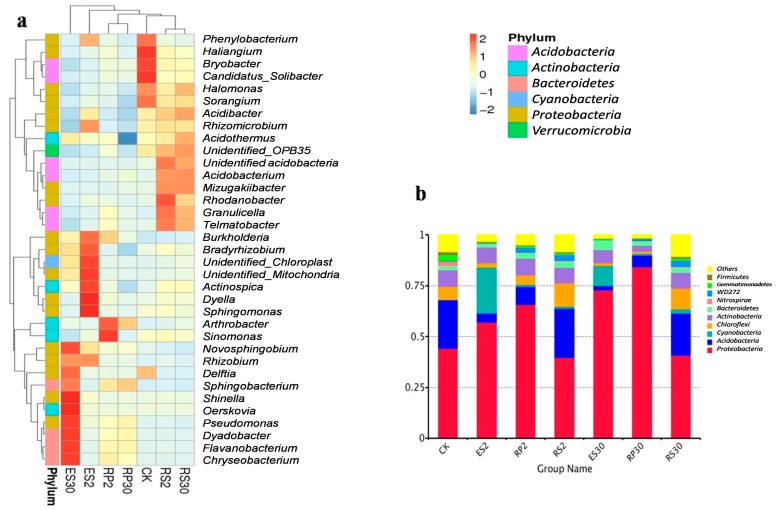
(**a**) Heat map showing the distribution of the 35 most abundant genera. (**b**) Distribution of the top 10 most abundant phyla in bulk soil (CK), rhizosphere of the two-year-old tea plantation (RS2), rhizoplane of the two-year-old tea plantation (RP2), endosphere of the two-year-old tea plantation (ES2), rhizosphere of the thirty-year-old tea plantation (RS30), rhizoplane of the thirth-year-old tea plantation (RP30) and endosphere of the thirty-year-old tea plantation (ES30).

According to rarefaction analysis, the number of Operational Taxonomic Units (OTUs) for 16S rRNA plateaued after 45,000 sequences at 97% similarity (Figure 2). This implied that the sequence depth was sufficient to relatively and accurately capture the diversity and richness of these samples. A total of 1,452,893 (average: 69,185) reads were analyzed by pyrosequencing. In total, 1,442,103 (average: 68,672) reads were paired successfully with 16S rRNA. After removing short and low-quality reads, singletons, replicates, and chimeras, 1,363,660 high quality reads (average: 64,936) were obtained from 21 samples. In total 1,210,023 tags (average: 67,223.5) were classified into 1,166,269 (average: 64,792.72) taxa tags. Based on ≥97% similarity, taxa tags were clustered into 54,719 (average: 2606) OTUs ranging from 1695 to 3684 per sample. In these taxa tags, 99.1% were classified as various bacteria, which primarily consist of 45 phyla, whereas the remaining 0.9% were classified as *Archaea* (Figure S2, Supplemental Dataset 1–5). Heat map analysis (Figure 3a) showed that the dominant bacterial phyla in bulk soil (CK) were *Proteobacteria (Phenylobacterium*, *Haliangium*, *Halomonas*, *Delftia*, and *Sorangium)* and *Acidobacteria (Bryobacter* and *Candidatus_solibacter).* In rhizosphere of the 30-year-old tea plantation (RS30) *Mizugakiibacter*, *Rodanobacter*, *Acidobacterium*, *Granulicella*, *Telmatobacter*, and *Verrucomicobia*, while in rhizosphere of the two-year-old tea plantation (RS2) *Halomonas*, *Acidobacter*, *Mizugakiibacter*, *Acidobacterium*, *Granulicella*, *Telmatobacter*, and *Acidothermus* genera were the most dominant. In rhizoplane of the two-year-old tea plantation (RP2) *Cyanomonas* and *Arthobacter*, while in rhizoplane of the 30-year-old tea plantation (RP30) *Arthobacter* and *Sphingomonas* were the most dominant genera. In endosphere of the two-year-old tea plantation (ES2) *Burkholderia*, *Bradyrhizobium*, *Dyella*, *Sphingomonas*, *Rhizobium*, *Cyanobacteria*, and *Actinospica* were dominant, while in endosphere of the 30-year-old tea plantation (ES30) *Novosphingobium*, *Pseudomonas*, *Shinella*, *Rhizobium*, *Delfia*, *Sphingobacterium*, *Dyadobacter*, *Flavanobacterium*, *Chryseobacterium*, and *Oerskovia* were the most dominant genera. The most dominant phyla across all the samples were *Proteobacteria*, *Acidobacteria*, *Cyanobacteria*, *Chloroflexi*, *Actinobacteria*, *Bacteroides*, *Nitrospirae*, *Verrucomicobia* (*WD272*), *Gemmatimonadetes*, and *Firmicutes* accounting for 90–96% of the bacterial sequences (Figure 3b).

### 2.4. Distinct and Overlapping Bacterial Communities of Root-Associated Bacterial Communities across Different Tea Plantations of Different Ages

**Figure 4:**
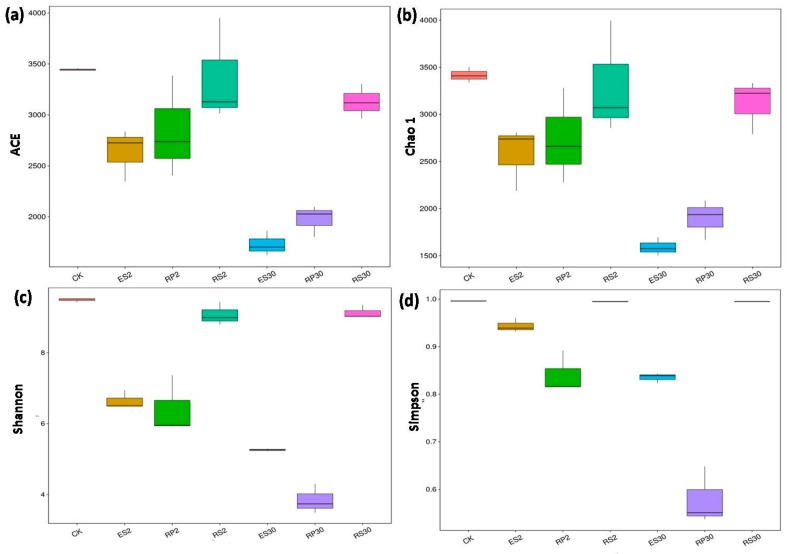
α-diversity indices including calculations of (**a**) abundance-based coverage estimators (ACE), (**b**) Chao1, (**c**) Shannon, and (**d**) Simpson in bulk soil (CK) and in various rhizo-compartments of different tea plantations of different ages: RS2, RP2, ES2, RS30, EP30 and ES30.

According to Chao1 and ACE estimators (Figure 4a,b), the rhizosphere, rhizoplane, and endosphere of the newly planted tea garden showed higher bacterial community richness than the continuous (30-year) monocropping tea field. Although the Shannon and Simpson diversity indices decreased significantly in the rhizoplane and endosphere of the continuous monoculture plants (30-year) when compared to the fresh tea plantation and the uncultivated field (Figure 4c,d), no significant differences were observed for the rhizosphere. Moreover, in the rhizo-compartments of the same type of tea garden, bacterial community richness showed a decreasing gradient from the rhizosphere toward the endosphere (Figure 4), but the alpha diversity in the same type of tea garden showed varying gradients. These results suggest that continuous monoculture could impact the variance in diversity and abundance of the bacterial communities in root-associated microbiomes within the samples.

**Figure 5:**
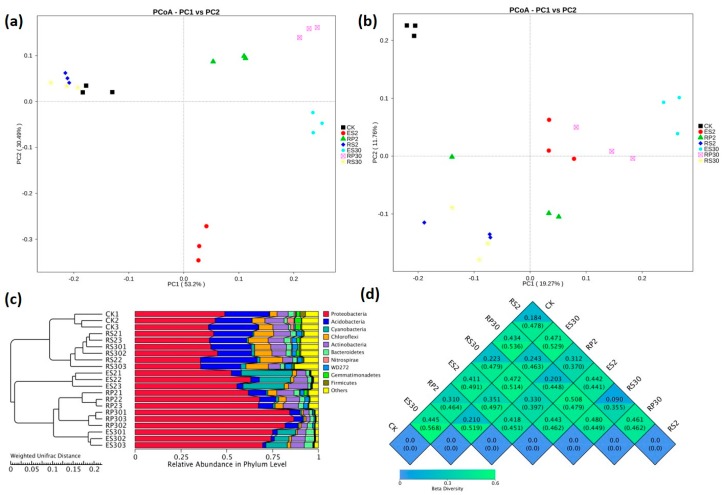
Biplot ordination of (**a**) weighted UniFrac principal coordinate analysis (PCoA) and (**b**) Unweighted UniFrac (UUF) principal coordinate analysis (PCoA) among various samples. CK represents a nearby uncultivated field, while RS2, RP2, and ES2 represent the rhizosphere, rhizoplane, and endosphere of the two-year tea plantation, and RS30, RP30, and ES30 represent the rhizosphere, rhizoplane, and endosphere of the 30-year-old tea plantation. (**c**) UPGMA/hierarchical clustering analysis based on weighted UniFrac distances showing the relative abundance of the most abundant bacterial phylum in various rhizo-compartments of different tea plantations of different ages. (**d**) β-diversity heat map based on weighted (WUF) and unweighted (UUF) UniFrac distances. Values in the upper and lower corners represented the WUF and UUF distances.

β-diversity was estimated by using both weighted (based on the abundance of taxa) and unweighted (sensitive to rare taxa) UniFrac distance matrices between samples. The weighted UniFrac principal coordinate analysis (PCoA) depicted that the bacterial communities in bulk soil and the rhizosphere were separated from the rhizoplane and endosphere and clustered along the principal coordinate axis-1, while the RP and ES were clustered along axis-2 (Figure 5a). Moreover, unweighted UniFrac PCoA analysis showed that the bacterial communities in the bulk soil and rhizosphere were different from the RP and ES and clustered along principle coordinate axis-1, and the RP and ES clustered along principle component axis-2 (Figure 5b).The unweighted pair group method with arithmetic mean (UPGMA) clustering analysis clearly indicated the differences in bacterial community structure among different samples, and similar results were observed for the same sample when analyzed in triplicate (Figure 5c). The weighted and unweighted UniFrac distances between CK and RS2 were 0.184 and 0.478, respectively and were 0.223 and 0.479 between CK and RS30, respectively. In comparison with CK and newly planted tea (RS2, RP2, and ES2) the weighted and unweighted UniFrac distances increased in the 30-year-old tea plantation (RS30, RP30, and ES30) (Figure 5d).

### 2.5. Interactions of Bacterial Abundance with Soil Physio-Chemical Properties and Root Exudates

**Figure 6:**
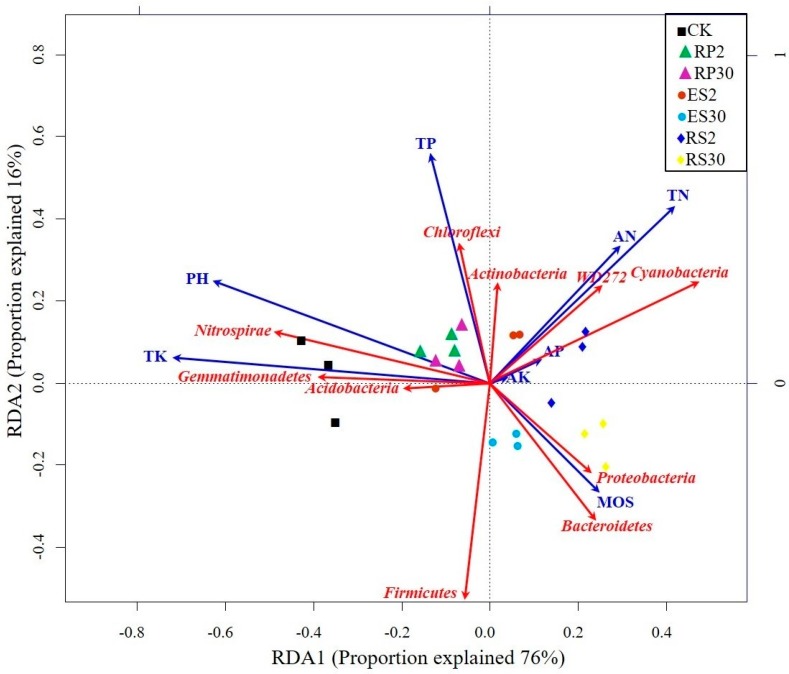
Redundancy analysis (RDA) Triplot (RDA on a covariance matrix) of the correlation between the most abundant phylum of bacteria and soil physiochemical properties, such as total phosphorus (TP), total nitrogen (TN), available nitrogen (AN), available potassium (AK), available phosphorus (AP), and pH across the various rhizo-compartments of the different tea plantations of different ages, where rhizosphere RS2 and RS30 are the rhizosphere, RP2 and RP30 are the rhizoplane, and endosphere ES2 and ES30 are the endosphere in two-year and 30-year-old tea gardens, respectively. The arrow length and direction correspond to the variance that can be explained by the environmental and response variables. The direction of an arrow indicates the extent to which the given factor is influenced by each RDA variable. The perpendicular distance between the abundance of bacterial phyla and environmental variable axes in the plot reflects their correlations. The smaller the distance, the stronger the correlation.

Redundancy analysis was performed to study the relationships between root exudates, soil physical and chemical properties, and the abundance of bacterial phylum. Strong associations were found among available nitrogen (AN), total nitrogen (TN), available potassium (AK), and available phosphorus (AP) with the abundance of *WD272*, *Cyanobacteria*, and *Actinobacteria*, clustering along axis-1 in the two-year-old tea plantation (Figure 6). Although most of the bacterial phylum depicted a strong negative correlation with the soil pH, the *Acidobacteria*, *Gemmatimonadetes*, and *Nitrospirae* had a strong positive association with soil pH, clustering along axis-2 in both the bulk soil and the 30-year-old tea plantation (Figure 6). The abundance of *Bacteroidetes* and *Proteobacteria* were found to be highly associated with soil moisture (MOS) in the 30-year-old tea plantation compared to the two-year-old tea plantation (Figure 6). However, *Chloroflexi* and *Actinobacteria* showed a strong positive association with the total phosphorus (TP), but were negatively associated with the soil moisture.

**Figure 7:**
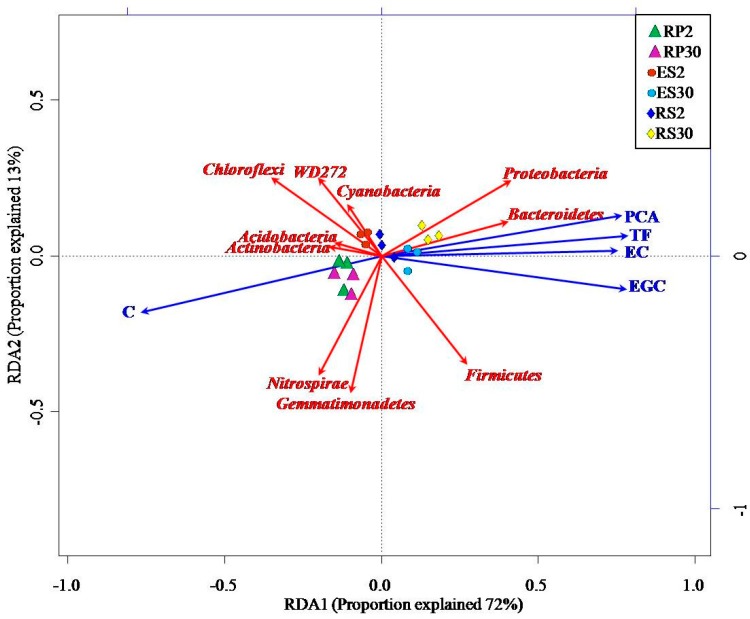
RDA Triplot (RDA on a covariance matrix) of the correlation between the most abundant phylum of bacteria and root exudates, such as C, EGC, EC, TF, and PCA, across various rhizo-compartments of different tea plantations of different ages, where the rhizosphere is represented by RS2 and RS30, the rhizoplane by RP2 and RP30, and the endosphere by ES2 and ES30, in two-year and 30-year-old tea gardens, respectively. The arrow length and direction correspond to the variance that can be explained by the environmental and response variables. The direction of the arrow indicates the extent to which the given factor is influenced by each RDA variable. The perpendicular distance between abundance of the bacterial phyla and the environmental variable axes in the plot reflects their correlations. The smaller the distance, the stronger the correlation.

Redundancy analysis (RDA) ordination between root exudates, allelechemicals, and bacterial phylum abundance depicted that the abundance of most bacterial phylum, except *Proteobacteria*, *Bacteroidetes* and *Firmicutes*, had a strong negative association with PCA, TF, EC, and EGC cluster along the first axis (RDA1) in the 30-year-old tea plantation (Figure 7). On the other hand, C was clustered along RDA axis-2 was strongly associated with most of the bacterial phyla (Figure 7). These results clearly indicate that the higher concentration of allelochemicals (PCA, TF, EC, and EGC) play a significant role in the declining bacterial communities in the 30-year-old tea plantation.

## 3. Discussion

The results shown here provide an insight for addressing the problems related to continuously growing a ratooning tea monoculture through the characterization of the microbiome, combined with the finer population structural details. Our design allowed us to successfully elaborate on the influence of a continuously monocultured tea plantation on its microbial community associated with all three rhizo-compartments. Moreover, this process also permitted us to reveal the extent to which plant-microbial interactions were mediated by the soil physio-chemicals and root exudates. Specifically, we characterized the microbial compositions of three distinct rhizo-compartments: the rhizosphere, rhizoplane, and endosphere, and showed the influence of external factors on each of these rhizo-compartments in bulk soil, on a fresh tea plantation, and an old tea plantation. Although these impacts were not significant, we found that the α- and β-diversity of microbes differ significantly across plantations of different ages. Depiction of RDA ordinations illustrated the association of bacterial phylum over time with the changing concentration of soil physiochemical properties and plant root exudates.

Recent studies on microbiomes have illustrated the power of deeper sequencing in describing the structural composition of plant microbiomes covering a geographical range of cultivation [40]. By adapting a deeper sequencing approach [35,41] most studies showed only the microbial abundance and impact of soil physiochemical properties on the microbiota present in the rhizosphere. However, the diversity and abundance of microbiota present in all three rhizo-compartments, and the influence of both soil physio-chemicals and root exudates, have not been previously studied together in for tea crops in plantations of different ages. To address this, we successfully adapted previously-described protocols for the removal of microbes from all three rhizo-compartments of new and old tea plantations. We found that the microbial communities, associated with rhizo-compartments under three field conditions, were chiefly clustered by the difference in the age of the tea plantations, which is in accordance with the previous work showing the effects of continuous plantation on soil-associated microbes [41,42]. However, the microbiota associated with the rhizosphere, rhizoplane, and endosphere in the same field did not show a significant difference. Peiffer et al. [42] also showed that field conditions did not affect the patterns of variations among the rhizo-compartments. Furthermore, we observed a greater α- and β-diversity of microbiota across plantations of different ages. These results suggest that the increasing age of the tea plants affects the microbiota in all three rhizo-compartments. Heat map analysis showed that most of the beneficial microbial genera, such as *Halomonas*, *Cyanomonas*, *Burkholderia*, *Bradyrhizobium*, *Dyella*, *Rhizobium*, *Cyanobacteria*, and *Actinospica* that are involved in the carbon, sulfur, and nitrogen cycles as well asprobiotics, decreased with the increasing age of the ratooning tea monoculture tea plantation. Zhao et al. [35] also showed that beneficial bacteria decreased and harmful bacteria increased with increasing age of *Pseudostellaria heterophylla* plantations. As the richness and β-diversity of microbiota clearly indicate differences across different-aged tea plantations, we hypothesized that these variations are chiefly governed-over time by the imbalance of soil physiochemical and/or root exudates in tea fields.

Our results showed no significant difference in the soil nutrients (N, P, and K).Only soil pH significantly decreased in the 30-year-old tea plantation when compared with the bulk soil and the two-year-old tea field, which resulted in lowering the abundance of rhizospheric microbiota, such as *Actinobacteria*, *Cyanobacteria*, *Chloroflexi*, and *WD272* [35], and showed direct association with *Nitrospirae*, *Gemmatimonadetes*, and *Acidobacteria.* On the other hand, *Proteobacteria* and *Bacteroidetes* were the most dominant bacterial group present in the 30-year-old tea plantation [43,44], which may be because these bacterial taxa prefer to live in a root vicinity with nutrient deficiency, high soil moisture (MOS), and lower pH. Previous studies on bacterial communities have clearly shown that nutrient deficiency did not significantly impact the soil microbiota, however soil pH was proven to be the most influential factor in shaping the soil microbiota [45,46,47]. The actual cause of a reduction in pH in older tea plantations was not studied until now, so we hypothesized that soil pH in the 30-year-old soil decreases with the increasing level of root exudates (allelochemicals).

Our results depicted higher levels of PCA, TF, EC, and EGC in ratooning 30-year-old tea monocropping fields, which may cause the lowering of soil pH over time [48]. In previous studies [32], C and EC were the carbon sources of some protobacteria, such as *Pseudomonas*, which either directly affected growth of the plant or indirectly affected it by biotransformation into more toxic compounds like TF and PCA. In our results, we showed that accumulation of these organic acids, such as PCA, could change the pH of the 30-year-old tea plantation over time. In the RDA study, the *Proteobacteria*, *Bacteroidetes*, and *Firmicutes* showed strong association with increasing levels of EC, EGC, TF, and PCA. Whereas C, which is secreted by the roots of young tea plants in significant amounts, could act as a raw source for catechin’s degradation of bacteria. For example, these results provide evidence that the rhizospheric microbiota in different aged tea plantations is influenced by pH and is indirectly affected by the carbon source-to-sink relationships within the plants [42]. In addition, these results suggested that the excessive accumulation of catechins can be prevented by removing the carbon sink sources, such as removal of fallen tea leaves and other plant debris from the soil.

In conclusion, the dynamic shift in microbiomes across a continuously monocultured tea plantation were related to root exudates. Moreover, soil pH changed with the changing level of root exudates (PCA), ultimately causing a decline in the population of pH-sensitive microbiota with continuous tea monocropping. Our findings suggest that removal of plant debris and pruned tea leaves from the soil can prevent the accumulation of PCA, TF, and EC, which may delay the reduction in soil pH. However, future research is needed to further explore the underlying mechanisms of rhizoshperic microbial interactions mediated by root exudates and to identify the contributing factors to soil sickness in continuous tea monocropping.

## 4. Materials and Methods

### 4.1. The Collection of Plant and Rhizosphere Soil Samples

Plant leaves, roots, and soil samples of tea were collected from freshly planted (2 years old) and old (30 years old) tea fields, and bulk soil was obtained from nearby uncultivated fields, located in the observation station in Anxi county (117°36′–118°17′ E, 24°50′–25°26′ N) on the south coast of Fujian province, China. These tea fields and nearby uncultivated tea fields shared the same environmental conditions and agronomic management. The sampling depth was about 30 cm in a range of about 25 cm from the root of the plant. Tea roots were carefully uprooted from the soil with a forked spade and slightly shaken to remove loosely attached soil. The rhizosphere soil tightly attached to the roots was brushed off and collected. The distance between the two plants was 0.5–0.7 m. The tea plants were 1.2 m in height and 0.9–1.2 m in width. At the time of sampling, the samples were taken at random, and each of the treatments included 15 rhizosphere soil samples. An ice box was used to bring the collected soil samples back to the laboratory. In order to reduce the error caused by spatial heterogeneity, five random sampling points within the 15 sampling sites were mixed into one soil sample, and three soil samples were obtained for each treatment [22]. To determine the microbial flora, we followed the method proposed by Edwards et al. [49] with little modification. Briefly, bacterial communities were derived from three different soil and tea plant compartments; the RS comprised of the soil tightly adhering to the root surface, the RP consisted of the suite of microbes present on the root surface by sonication, and the ES covered the interior of the same plant roots after sonication. A five-point sampling method was used [13] to make one sample with three replicates. All the samples were stored at −80 °C.

### 4.2. Quality Parameters and Soil Properties Determination

Quality parameters such as the anine (TNN), theophylline (TPY), total polyphenols (TPP), and total free amino acids (TAA) of tea plantations of different ages were determined by the method of Peng et al. [50]. Soil pH was determined using a glass electrode pH meter (1:2.5 soil to water suspensions). Soil moisture (MOS) and water content (WC) of tea leaves were measured by the following Oven Drying Method. Total Potassium (TK), TN and TP were measured by the NaOH melt flamer method, dichromate oxidation, and Kjeldahl digestion respectively [51]. AP was extracted by using ammonium fluoride and hydrochloric acid and measured by the following Molybdenum Blue Method. AN was calculated by the alkaline hydrolysable method. AK was extracted using ammonium acetate and determined by flame photometry [52].

### 4.3. Identification and Quantification of Allelochemicals from Tea Root Exudates

To identify and quantify allelochemical concentration in tea roots exudate samples, ≥98% pure C, ECG, EGC, EGCG, EC, PCA, and TF were purchased from Cayman Chemical (1180 E. Ellsworth Road, Ann Arbor, MI, USA) as external standards. HPLC–MS grade methanol and formic acid were purchased from Sigma-Aldrich (St. Louis, MO, USA). Firstly, the stock solutions were prepared by dissolving 1–2 mg of each standard in 1–2 mL of solution containing 99.9% methanol and 0.1% acetic acid by volume. To obtain the calibration curves, serial dilutions were performed using the same solvent as used to prepare the stock solutions at different concentrations ranging between 1.25–10 µg/mL. A 10-µL aliquot of all solutions were injected for HPLC–ESI–MS analysis. Good linearity over the calibration range was achieved with all coefficients of correlation >0.998. The concentration of catechins from roots and soil was determined by following the method of Wang et al. [32] with slight modifications. Ten grams of soil, and 5 grams of roots and leaves were vortexed in a 15-mL solution containing 0.1% acetic acid and 50% methanol by volume at 150 rpm for 24 h. The sample was then centrifuged for 10 min at 4500 rpm and supernatant fluid was moved to a sample collection vial to perform liquid chromatography (LC). Following this, HPLC–ESI–MS was carried out using T3 RP-18 column (100 × 2.1 mm; 5 µm; Waters, Milford, MA, USA) eluted with buffer A (Sigma-Aldrich Co., St. Louis, MO, USA) (0.1% acetic acid) and buffer B (EMD Milliopre Corporation, Billerica, MA, USA) (100% methanol) at a flow rate of 300 µL/min at 25 °C. Initially, the column was eluted with 95% buffer B, followed by a linear increase in buffer A to 35% from 0–10 min, and further maintained in 90% buffer A for 5 min. Then, a linear increase in buffer B to 95% was maintained. Finally, the column was maintained in 95% buffer B for 8 min. The total time for running one sample was 19 min. The negative ionization mode was selected to perform mass spectrometry at a temperature of 100 °C, and ion scans were carried out at low-energy collision (20 eV) using nitrogen as the collision gas. All the data from HPLC–ESI–MS were processed to determine the mean concentrations of the selected catechin compounds in each sample, by using Bruker Daltonics Data analysis software version 4.0 (Thermo Fisher Scientific, Waltham, MA, USA).

### 4.4. DNA Extraction and Purification

Whole genome DNA was extracted from bulk soil, rhizosphere, rhizoplane, and endospheric samples by using Fast DNATM Spin Kit, specialized for extracting DNA from soil, following the manufacturer’s instructions manual (MP Biomedical, Santa Ana, CA, USA). Then all the DNA samples were subjected to gel electrophoresis and further purification, using Universal DNA Purification Kits according to the manufacturer’s instructions (Tiangen Biotech Co., Ltd., Beijing, China). DNA was quantified by using Nanodrop (Thermo Fisher Scientific, Waltham, MA, USA) before being stored at −20 °C for further molecular analysis.

### 4.5. The Metagenomic Analysis of the Root-Associated Rhizo-Compartment Bacteria

Purified DNA samples were sent to Novogene Bioinformatics Technology Co., Ltd. (Beijing, China) to determine the bacterial community structure. Prior to high throughput sequencing, 16S V4, a distinct gene region of 16S rRNA, was amplified using specific primer 515F-806R with barcodes [53]. All PCR reactions were conducted using 30 µL total reaction volume with 15 µL of Phusion^®^ High-Fidelity PCR Master Mix (New England Biolabs (Beijing) Ltd., Beijing, China) containing ~10 ng template DNA and 0.2 µM of each primer pair. The PCR condition set was denaturation at 98 °C for 1 min, followed by 30 cycles of denaturation at 98 °C for 10 s, annealing at 50 °C for 30 s, and elongation at 72 °C for 60 s, with a final extension at 72 °C for 5 min. Then electrophoresis using 2% agarose gel solution was performed to verify the successful DNA amplification mixing PCR products with the same amount of 1× loading buffer containing SYB green (TIANGEN, Beijing, China). Samples showing main strip brightness ranging between 400–450 bp was selected for further sequencing. PCR products were purified by using Gene JET Gel Extraction Kit (Qiagen, Hilden, Germany) prior to sequencing. Sequencing libraries were created in Illumina using specialized NEB Next^®^ Ultra™ DNA Library Prep Kit (New England Biolabs (Beijing) Ltd., Beijing, China) according to the manufacturer’s instructions and index codes were added. The quality of the developed sequencing library was checked in both the Agilent Bioanalyzer 2100 system (Agilent, Santa Clara CA, USA) and the Qubit^®^ 2.0 Fluorimeter (Thermo Fisher Scientific, Waltham, MA, USA). Finally, 250/300bp paired-end reads were generated on an Illumina MiSeq platform (Illumina, San Diego, CA, USA).

### 4.6. Statistical and Bioinformatics Analysis

Raw sequences were classified according to the specific barcode assigned to each sample, using Quantitative Insights Into Microbial Ecology (QIIME) (CO, USA) [53]. Paired-end reads were merged from the original DNA segments using FLASH (Baltimore, MD, USA) [54]. Paired-end reads were assigned to each sample according to the unique barcodes attached with DNA fragments. Sequences were analyzed using UPARSE-OTU and UPARSE-OUT reference algorithms with UPARSE software package (CA, USA). Alpha (within samples) and beta diversity (among samples) were calculated using QIIME (CO, USA) [53]. The same Operational Taxonomic Units (OTUs) were assigned to the sequences with ≥97% in each sample. One representative sequence was selected for each OTU to annotate the taxonomic information of each representative sequence by using the RDP classifier.

To measure the Alpha diversity within the sample, we rarified the OUT table and then four diversity matrices were calculated: Chao1 estimates the species abundance, and the Observed Species, Simpson, and Shannon indices were used to determine the community diversity. Moreover, rarefaction curves were developed for each of these four indices. Abundance of each bacterial taxa, from phylum to species, was shown graphically using a Krona Chart. Beta diversity (among samples) was measured for both weighted and unweighted UniFrac distances using QIIME (Version 1.7.0) (CO, USA). Principal Component Analysis (PCA) and Principal Coordinate Analysis (PCoA) were performed and visualized using R (Version 2.15.3) packages; stat, WGCNA and ggplot2 (Elegants graphics for data analysis, New York, NY, USA). We identified the association of the most abundant bacterial phylum with selected soil physiochemical properties and root exudates using partial-RDA [55], and triplots were generated using vegan and ggplot2 packages in R software (R version 3.3.1) (Foundation for Statistical Computing, Vienna, Austria).

## Figures and Tables

**Table 1:** Quality parameters of tea leaves from tea plantations of different ages. TNN, TPY, TPP, TAA, and WC represent the anine, theophylline, total polyphenols, total free amino acids, and water content, respectively. 2YTL and 30YTL represent the two-year-old tea field and the 30-year-old tea field leaves, respectively. Different letters (^a^ and ^b^) in columns show a significant difference determined by Tukey’s test (*p* ≤ 0.05, *n* = 2).

Treatments	TNN (g/kg)	TPY (mg/kg)	TPP (g/kg)	TAA (g/kg)	WC (%)
2YTL	1.70 ^a^	0.12 ^a^	106.40 ^a^	12.44 ^a^	0.76 ^a^
30YTL	0.47 ^b^	0.02 ^b^	30.66 ^b^	8.87 ^b^	0.56 ^a^

## Supplementary Materials

Supplementary materials can be found at www.mdpi.com/1422-0067/18/8/1727/s1.
