# Supplementary material for: Spatial Distribution Patterns of Root-Associated Bacterial Communities Mediated by Root Exudates in Different Aged Ratooning Tea Monoculture Systems
Source: Int J Mol Sci. 2017 Aug 8;18(8):1727. doi: 10.3390/ijms18081727 (PMC5578117; doi:10.3390/ijms18081727)
Supplement: Supplementary file 1 [file ijms-18-01727-s001.zip › ijms-207312-final-supplementary materials.pdf]

Article

Spatial Distribution Patterns of Root-Associated Bacterial Communities Mediated by Root Exudates in Different Aged Ratooning Tea Monoculture Systems

Yasir Arafat [1,2,3,4], Xiaoya Wei [1,2,3], Yuhang Jiang [1,2,3,4], Ting Chen [1,2,3,4], Hafiz Sohaib Ahmed Saqib [4], Sheng Lin [1,2,3,4,*] and Wenxiong Lin [1,2,3,4,*]

1. Key Laboratory of Fujian Province for Agroecological Process and Safety Monitoring, Fujian Agriculture and Forestry University, Fuzhou 35002, China; arafat_pep@yahoo.com (Y.A.); xlwei112930@163.com (X.W.); janmiky@163.com (Y.J.); iamchenting@126.com (T.C.)
2. Key Laboratory of Ministry of Education for Crop Genetics/Breeding and Integrative Utilization, Fujian Agriculture and Forestry University, Fuzhou 35002, China
3. College of Life Science, Fujian Agriculture and Forestry University, Fuzhou 35002, China
4. Institute of Agroecological Ecology, Fujian Agriculture and Forestry University, Fuzhou 35002, China; sohaibsaqib@gmail.com

* Correspondence: linsh@fafu.edu.cn (S.L.); lwx@fafu.edu.cn (W.L.); Tel.: +86-0591-837-789301

Table S1. Physio-chemical properties of soil from different years ratooning tea fields. AN, TP, AP, TK, AK, MOS represents total nitrogen, available nitrogen, total phosphorus, available phosphorus, total potassium, available potassium, soil moisture respectively. CK, 2RS, 30RS represent the bulk soil nearby uncultivated field, newly planted 2-year tea field and 30-year monoculture tea field respectively. Different letters (a and b) in columns show significant difference determined by Tukey's test ($p \leq 0.05$, $n = 3$).

Treatments	TN(g/kg)	AN(mg/kg)	TP(g/kg)	AP(mg/kg)	TK(g/kg)	AK(mg/kg)	MOS	pH
CK	0.53^{b}	13.23^{b}	13.06^{a}	93.33^{a}	10.42^{a}	127.21^{ab}	19.50^{b}	5.04^{a}
2RS	1.02^{a}	14.84^{a}	16.64^{a}	95.00^{a}	10.91^{a}	123.72^{b}	18.89^{b}	4.18^{b}
30RS	0.99^{a}	13.91^{ab}	16.16^{a}	98.33^{a}	10.93^{a}	134.34^{a}	21.85^{a}	3.30^{c}

Table S2. The concentration and percentage recovery of allelochemicals in newly planted two-year (*2ys*) and 30-year (*30ys*) consecutive monoculture tea root exudates. PCA, EGC, EGCG, EC, C, ECG and TF represents protocatechuic acid, epigallocatechin, epigallocatechin gallate, epicatechin, (+)-catechin, epicatechin gallate and taxifolin respectively. Different letters (a and b) in columns show significant difference determined by Tukey's test ($p \leq 0.05$, $n = 7$), ± standard error.

Autotoxins	Calibration curve	Recovery (%)		Concentration (mg/kg)	
		2ys	*30ys*	2ys	30ys
PCA	y=99,808x + 57,585	100.97 ± 1.69^{ab}	108.74 ± 9.87^{a}	2.04^{b} ± 1.04	14.58^{a} ± 1.04
EGC	y=79,851x + 1557	98.33 ± 6.24^{ab}	97.68 ± 11.91^{a}	1.73^{b} ± 0.17	3.20^{a} ± 0.17
EGCG	y=14,749x - 2599	89.48 ± 2.36^{a}	52.31 ± 10.71^{b}	6.95^{a} ± 0.75	3.27^{b} ± 0.75
EC	y=13,123x + 7893	71.05 ± 24.34^{b}	102.67 ± 3.09^{a}	11.98^{b} ± 1.69	21.38^{a} ± 1.69
C	y=42,873x - 6220	55.71 ± 7.62^{c}	60.00 ± 7.44^{b}	21.31^{a} ± 1.19	3.70^{b} ± 1.19
ECG	y=31,166x + 74,903	74.69 ± 1.38^{bc}	69.90 ± 2.16^{b}	1.18^{b} ± 0.27	2.62^{a} ± 0.27
TF	y=71,649x + 55,050	51.49 ± 13.10^{c}	52.36 ± 14.31^{b}	1.61^{b} ± 0.14	3.65^{a} ± 0.14

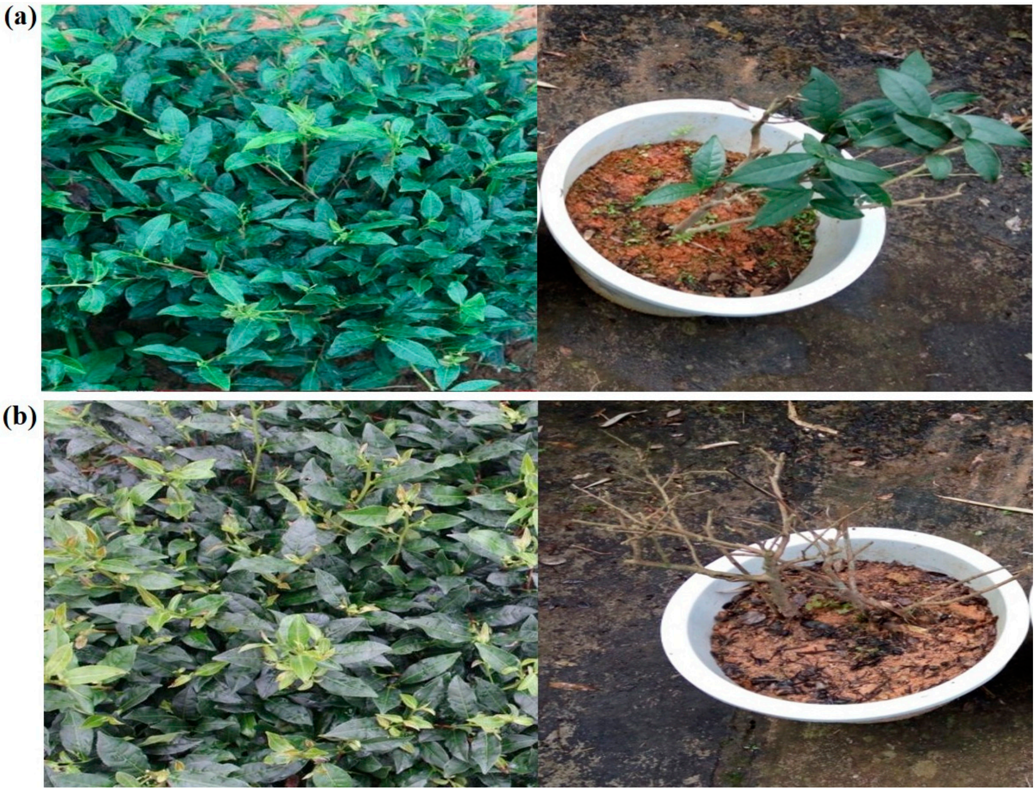

Figure S1. Replanting/ratooning problems in different years' tea gardens soil. (**a**) Ratoon tea seedling in fresh soil and (**b**) Ratoon tea seedling in old tea soil (30-year), under same field conditions.

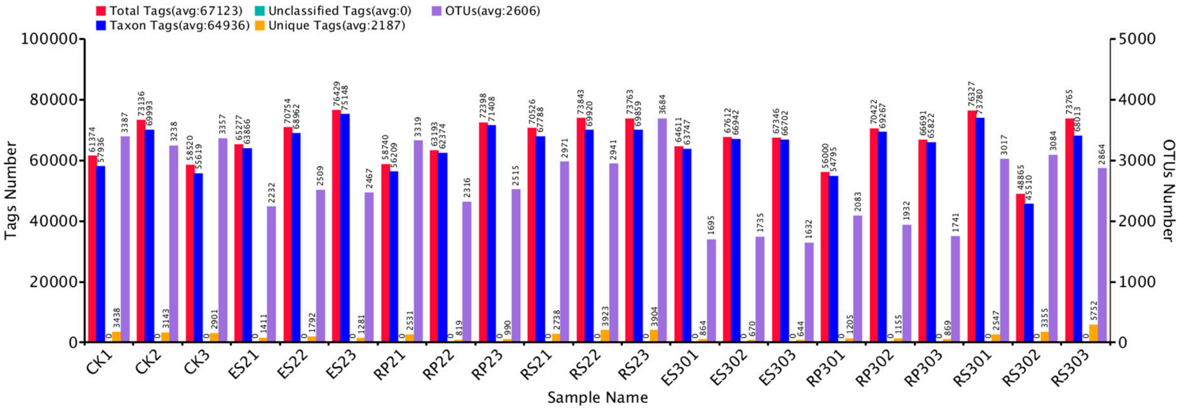

Figure S2. Statistics of Operational Taxonomic Units (OUT) cluster and species annotation for individual samples. Total Tags (red columns): represent the total numbers of effective tags; Unique Tags (orange columns): represent the total numbers of singletons which were removed from the dataset before further analysis; Taxon Tags (blue columns): represent the total numbers of tags subjected to OTU cluster and with species annotation; Unclassified Tags (green columns): represent the total numbers of tags without species annotation; OTUs (purple columns): represent the OTUs numbers for each sample. Bulk, RS2, RP2, ES2, RS30, RP30, ES30 represent the control with no *Camellia sinensis* (L.) cultivation, rhizosphere, rhizhoplane and endosphere of the newly planted, two-year and thirty-year monoculture plots, respectively.
